# DegS protease regulates antioxidant capacity and adaptability to oxidative stress environment in *Vibrio cholerae*

**DOI:** 10.3389/fcimb.2023.1290508

**Published:** 2023-11-20

**Authors:** Kaiying Wang, Huifang Lu, Mei Zou, Guangli Wang, Jiajun Zhao, Xiaoyu Huang, Fangyu Ren, Huaqin Hu, Jian Huang, Xun Min

**Affiliations:** ^1^Department of Laboratory Medicine, Affiliated Hospital of Zunyi Medical University, Zunyi, Guizhou, China; ^2^School of Laboratory Medicine, Zunyi Medical University, Zunyi, Guizhou, China

**Keywords:** *Vibrio cholerae*, DegS protease, antioxidation, cAMP-CRP, XthA

## Abstract

Adaptation to oxidative stress is critical for survival of *Vibrio cholerae* in aquatic ecosystems and hosts. DegS activates the σ^E^ envelope stress response. We have previously revealed that DegS may be involved in regulating the oxidative stress response. In this study, we demonstrated that deletion of the *degS* gene attenuates the antioxidant capacity of *V. cholerae*. In addition, our results further revealed that the regulation of antioxidant capacity by DegS in *V. cholerae* could involve the cAMP-CRP complex, which regulates *rpoS*. XthA is an exonuclease that repairs oxidatively damaged cells and affects the bacterial antioxidant capacity. qRT-PCR showed that DegS, σ^E^, cAMP, CRP, and RpoS positively regulate *xthA* gene transcription. XthA overexpression partially compensates for antioxidant deficiency in the *degS* mutant. These results suggest that DegS affects the antioxidant capacity of *V.cholerae* by regulating *xthA* expression via the cAMP-CRP-RpoS pathway. In a mouse intestinal colonization experiment, our data showed that *V.cholerae degS*, *rpoE*, and *rpoS* gene deletions were associated with significantly reduced resistance to oxidative stress and the ability to colonize the mouse intestine. In conclusion, these findings provide new insights into the regulation of antioxidant activity by *V.cholerae* DegS.

## Introduction

Oxidative stress caused by excess reactive oxygen species (ROS) or a reduction in antioxidant capacity poses a significant threat to bacteria (Sudharsan et al., 2022). ROS, primarily superoxide anions, hydroxyl radicals, and hydrogen peroxide, are highly reactive substances produced as a result of the incomplete reduction of oxygen or redox production of chemicals (Si et al., 2015; Seixas et al., 2021). ROS can damage biomolecules such as DNA, RNA, proteins, and lipids (Chautrand et al., 2022). Hence, bacteria have developed antioxidant systems to cope with oxidative stress and ensure their survival and pathogenicity (Mishra and Imlay, 2012). For example, *Escherichia coli* utilizes OxyR and SoxRS to sense ROS signals and subsequently coordinate the expression of a set of genes encoding ROS-scavenging enzymes such as catalase and peroxidase (Honn et al., 2017).

*Vibrio cholerae* is an intestinal pathogen that lives in aquatic environments and is classified into more than 200 serogroups based on *V.cholerae* cell surface O antigens (Kaper et al., 1995; Clemens et al., 2017). Non-O1/non-O139 *V.cholerae* strains (NOVC) have been linked to sporadic localized cholera-like gastrointestinal diseases such as abdominal pain and diarrhea, as well as extra-intestinal infections like sepsis, infectious shock, pneumonia, and meningitis (Maraki et al., 2016; Chen et al., 2020). Despite the rise in NOVC infection cases, there has been little research on NOVC adaptability to oxidative stress. Antioxidant capability is critical for the survival of *V.cholerae in vivo* and *in vitro*. Therefore, it is of great significance to study the adaptability of non-O1/non-O139 *V.cholerae* under oxidative stress. *V.cholerae* can cause severe diarrhea and vomiting in humans when food contaminated with *V. cholerae* is ingested (Clemens et al., 2017; Qin et al., 2020; Wang et al., 2022). Diarrhea facilitates the passage of *V.cholerae* from the human body into the aquatic environment, where it can reinfect the human body. During this cycle, *V.cholerae* senses a variety of environmental signals and responds by adjusting gene expression to adapt effectively to its new ecological niche. Oxidative stress is one of the main stresses that must be overcome when *V. cholerae* infects the host and in the external environment (Qadri et al., 2002; Bhattacharyya et al., 2004; Lesser, 2006; Muller et al., 2017). ROS levels in the stools of patients with cholera are considerably higher than those in normal individuals (Wang et al., 2017). Duodenal samples from patients with cholera showed that oxidative stress-related proteases were activated (Ellis et al., 2015) and that *V.cholerae* isolated from the slop-like stools of cholera patients displayed greater tolerance to ROS (Qadri et al., 2002). The host produces large amounts of ROS to combat *V.cholerae* invasion and colonization, while *V.cholerae* regulates the expression of antioxidative stress genes to adapt to the host environment (Wang et al., 2017). For example, OxyR is critical for hydrogen peroxide resistance in *V.cholerae*, whereas OhrR, a regulator of *ohrA*, regulates the susceptibility of *V.cholerae* to organic hydrogen peroxide (Wang et al., 2012; Liu et al., 2016). Consequently, antioxidant activity is vital for the survival and pathogenicity of *V.cholerae* in aquatic environments and humans. From the perspective of cholera control, it would be beneficial to explore the details of antioxidant regulation in *V.cholerae* and establish methods to weaken this response.

DegS is a serine protease located in the bacterial periplasm that strictly controls the σ^E^ (*rpoE*) stress-response pathway (de Regt et al., 2015). In *Salmonella*, the *rpoE* mutant shows significantly increased sensitivity to H_2_O_2_ (Testerman et al., 2002; Hews et al., 2019). Our previous RNA sequencing results showed that *degS* knockout led to the downregulation of genes involved in the oxidative stress response. Gene-Act network analysis indicated that the cAMP-CRP-RpoS pathway is suppressed in *degS* knockout strains (Huang et al., 2019). The cAMP-CRP-RpoS pathway plays an essential role in oxidative stress in *Salmonella* (Cheng and Sun, 2009). Our previous results showed that DegS regulated *V.cholerae* motility and chemotaxis via cAMP–CRP–RpoS–FlhF pathway(Zou et al., 2023).In the present study, we investigated the effect of DegS on the antioxidant capacity of *V.cholerae*. Our results show that DegS regulates the expression of the nucleic acid exonuclease III gene *xthA* through the cAMP-CRP-RpoS pathway, thereby affecting the antioxidant capacity of *V.cholerae.*

## Materials and methods

### Strains and culture conditions

*V.cholerae* HN375 strain is a non-O1/non-O139 strain obtained from the China Type Culture Collection Center (CCTCC AB209168) (Luo et al., 2011) and was the wild type (WT) in this study. *Escherichia coli* DH5α was used for cloning and *WM3064* was used as donor bacteria in the conjugation transformation experiment. All strains were cultured on Luria Bertani (LB) medium. In the killing assay and survival under starvation stress assay, using AKI medium contains 1.5% Bacto-peptone, 0.4% yeast extract, 0.5% NaCl, and 0.3% NaHCO_3_. 100μg/mL ampicillin or 50μg/mL chloramphenicol were added to LB or AKI medium if necessary. All bacterial strains and plasmids used in this study are listed in **Supplementary Table 1**.

### DNA manipulation and gene technology

All deletion mutants were constructed using the suicide plasmid pWM91 in the wild-type HN375 strain (Wu et al., 2015). The primers used are listed in **Supplementary Table 2**. To construct complementary mutants, the full-length coding regions of *cyaA*, *crp*, *rpoS*, and *xthA* were cloned into the pBAD24 vector and transformed by electroporation into *ΔdegS* strains named *ΔdegS*+pBAD24-*cyaA*, *ΔdegS*+pBAD24-*crp*, *ΔdegS*+pBAD24-*rpoS*, and *ΔdegS*+pBAD24-*xthA*. To obtain double overexpression strains, the full-length coding region of *crp* was cloned into the pBAD33 vector and transformed by electroporation into a *ΔdegS*+pBAD24-*cyaA* high expression strain named *ΔdegS*+pBAD24/33-*cyaA/crp* (Sun et al., 2014). All complementary and overexpression strains were cultured in LB medium containing 0.1% arabinose to induce gene expression.

### Quantitative real-time quantitative polymerase chain reaction (qRT-PCR)

All strains were grown in LB liquid medium until the middle of the exponential period, reaching an OD_600_ of 0.6. Total RNA was extracted using the TRIzol reagent. qRT-PCR assays were performed as previously described using 16S RNA as an internal reference gene (Huang et al., 2019). 16sRNA expression was also assessed in each group (**Supplementary Figure 1**). All experiments were repeated three times.

### Hydrogen peroxide (H_2_O_2_) and cumene hydroperoxide (CHP) disk diffusion assay

The bacterial concentration was adjusted to to 1×10^8^ CFU/mL at the middle logarithm and 100μL of bacterial solution was taken for plate coating. Six sterile circular filter paper plates were placed flat on the surface of LB medium. Using a pipette to 10μL containing 0 (control), 30, 50, 100, 200, and 300 mmol/L H_2_O_2_ and 2μL containing 0 (control), 30, 50, 100, 200, and 300 mmol/L CHP were added dropwise onto sterile circular filter paper plates and incubated overnight at 37°C, and the diameter of the inhibition circle was measured by vernier calipers (Wang et al., 2012). All experiments were repeated three times.

### Hydrogen peroxide (H_2_O_2_) and cumene hydroperoxide (CHP) killing assay

The strains were cultured on LB plates at 37°C overnight. They were normalized to an OD_600_ of 1.0 in liquid medium. A 1:1,000 dilution of normalized culture was used to inoculate fresh AKI medium, incubated at 37°C for 2 hours with standing and 2h with shaking, and treated with final concentrations of 1, 5, 10, 20, and 30 mmol/L H_2_O_2_ and CHP for 2 hours. Gradient dilutions were performed and the cells were counted on LB plates. The survival rate of each bacterium was calculated by comparing the number of colonies with those of samples not treated with H_2_O_2_ and CHP (Wang et al., 2017). All experiments were repeated three times.

### Survival under starvation stress assay

These strains were cultured overnight on LB plates at 37°C and normalized to 10^9^ in fresh liquid medium with an OD600 of 1.0. A 1:1000 dilution of the normalized culture was used to inoculate the fresh AKI medium, which was then placed in an anaerobic bag for incubation. At the start of the anaerobic incubation, we used an anaerobic indicator to test the anaerobic incubation conditions with no oxygen, and the anaerobic incubation was done at 37°C for 4 hours and incubated overnight at 22°C in artificial seawater. To create artificial seawater, 40.0g of sea salt is dissolved in 1 liter of sterilized ddH_2_O. Dilution spot plates were sampled every 24 hours on LB plates. The survival rate of each bacterium was calculated by comparing the number of colonies with that of the non-anaerobically cultured colonies (Wang et al., 2017). All experiments were repeated three times.

### Mouse intestinal colonization assay

An intestinal colonization model was established using 5- to 6-week-old CD1 mice. All animal experiments conducted in this study were approved by the Ethics Committee of the Affiliated Hospital of Zunyi Medical University. One hundred forty-four mice were randomly divided into two groups according to sex, with 72 mice in each group. One group was treated with 1% N-acetylcysteine (Amrouche-Mekkioui and Djerdjouri, 2012) and 0.4% aspartame (NAC+) in drinking water, while the other group was treated with 0.4% aspartame (NAC-) in drinking water for 1 week. Subsequently, the two groups of mice were randomly divided into nine groups (n=8). The mice were fed sterile water containing streptomycin (5 mg/mL) from the day before the experiment until the end of the experiment. Streptomycin pretreatment of adult mice allows for some intestinal ROS production and inhibits the colonization ability of normal intestinal host bacteria in the mice intestinal tract, allowing for better colonization of the mice gut by *V.cholerae* (Spees et al., 2013; Liu et al., 2016). After the strains were cultured to the exponential growth phase, the cells were collected by centrifugation at 1200rpm for 3 min. The bacterial precipitates were washed twice with 1×phosphate-buffered saline, and the bacterial suspension was adjusted to an OD_600_ of 0.5, approximately 1.0×10^8^ CFU/mL. Each mouse was gavaged with 100μL (1×10^7^CFU) of the cell suspension, and 100μL of 1×phosphate-buffered saline was used as a negative control. The inoculum dose was determined by pre-experimental exploration, and it was found that a high concentration (1×10^8^ CFU per 100μL of bacterial solution) resulted in massive mortality in mice within 18 hours, whereas 1×10^7^CFU per 100μL of bacterial solution had no significant effect. The mice were euthanized 18hours after gavage, and small intestinal tissues were dissected, weighed, and homogenized. The 100μL bacterial solution was diluted and coated onto LB plates containing streptomycin 0.01 mg/mL. The number of colonies was counted after overnight incubation and expressed as the logarithm of the number of colonies formed per gram of intestine (CFU/g).

### Statistical analysis

Differences between two groups were analyzed using unpaired two-tailed *t*-tests, differences between multiple groups using one-way ANOVA, and multiple comparison tests using Bonferroni analysis, with *P*<0.05 considered statistically different for comparisons between groups. All experiments were performed on at least three independent replicates, and all values are expressed as mean ± SD.

## Results

### DegS enhances the antioxidant capacity of *V. cholerae*

**Figure 1:**
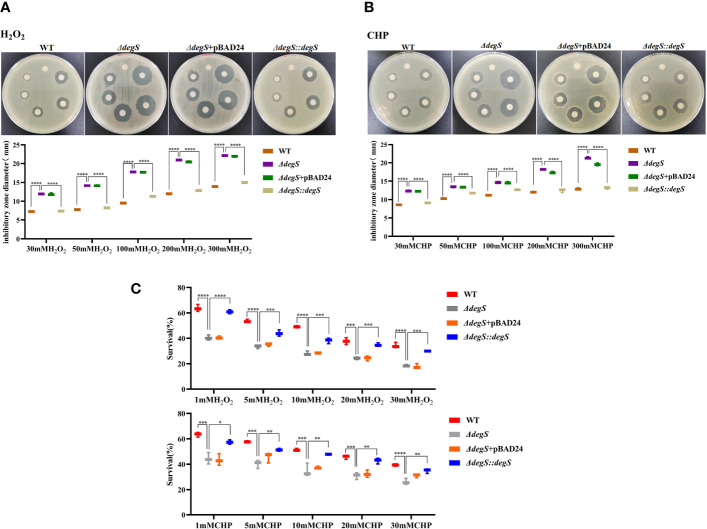
Effects of DegS on the antioxidant capacity of *V. cholerae*. **(A, B)** Sterile paper disk with different concentrations of H_2_O_2_ and CHP were placed on the corresponding strain plates for overnight incubation, with the oxidant concentrations ranging from small to large (0, 30, 50, 100, 200, and 300 mM) in a counterclockwise direction, and the diameter of the zone of inhibition was measured for each strain. **(C)** The killing assays were performed with 1, 5, 10, 20, and 30 mM H_2_O_2_ and CHP on the corresponding strains, and the survival rate of each strain was calculated by comparing the CFU of the treated and untreated samples. Data are means and standard deviations of three replicate experiments and were analyzed using one-way analysis of variance (ANOVA). *, *P*<0.05; **, *P*<0.01; ***, *P*<0.001; ****, *P*<0.0001.

Our previous GO analysis of differential genes suggested that the genes downregulated after *degS* knockout were mainly involved in processes such as oxidative stress (Huang et al., 2019), implying that DegS may affect the oxidative stress response in *V. cholerae.* To verify this hypothesis, we performed a disk diffusion assay to determine the resistance of the WT and *degS* mutants to different concentrations of hydrogen peroxide (H_2_O_2_) and cumene hydroperoxide (CHP). These results showed that the diameter of the inhibition zone of the *degS* mutant was significantly larger than that of the WT after treatment with both oxidants and that the inhibition zone of the revertant strain *ΔdegS::degS* was restored to a diameter close to that of the WT strain (**Figures 1A, B**). The pBAD24 empty plasmid did not restore the antioxidant capacity of *ΔdegS*. Additionally, we used different concentrations of H_2_O_2_ and the CHP killing assay, and the results showed that a significantly reduced survival rate of *ΔdegS* compared with that of the WT strain; the survival rate of *ΔdegS::degS* was partially restored, and the pBAD24 empty plasmid had no restorative effect (**Figure 1C**). These results indicate that the *degS* mutation leads to a reduction in the resistance of the non-O1/non-O139 *V. cholerae* strain HN375 to H_2_O_2_ and CHP.

### The positive regulation of *V. cholerae* antioxidant capacity by DegS is partially through σ^E^

**Figure 2:**
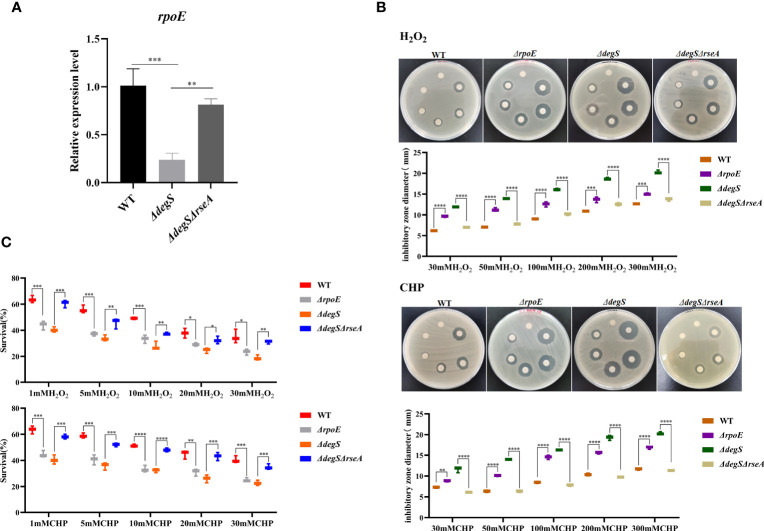
DegS regulates the antioxidant capacity of *V. cholerae* through σ^E^. **(A)** Comparison of the relative expression levels of *rpoE* genes in different strains with 16sRNA as an internal reference gene (unpaired *t*-test). **(B)** Sterile paper disk with different concentrations of H_2_O_2_ and CHP were placed on the corresponding strain plates for overnight incubation, with the oxidant concentrations ranging from small to large (0, 30, 50, 100, 200, and 300 mM) in a counterclockwise direction, and the diameter of the zone of inhibition was measured for each strain. **(C)** The killing assays were performed with 1, 5, 10, 20, and 30 mM H_2_O_2_ and CHP on the corresponding strains, and the survival rate of each strain was calculated by comparing the CFU of the treated and untreated samples. Data are means and standard deviations of three replicate experiments and were analyzed using one-way analysis of variance (ANOVA). *, *P*<0.05; **, *P*<0.01; ***, *P*<0.001; ****, *P*<0.0001.

DegS is the initiator of the σ^E^ stress response pathway and plays an important role in the regulation of stress response in *E. coli* (Bongard et al., 2019). RseA binds to σ^E^ as an anti-σ factor, preventing its release, and when the DegS protease is activated, the periplasmic structural domain of RseA is cleaved by DegS, thereby releasing σ^E^ to regulate the transcription of related stress-responsive genes (Kim, 2015). To investigate whether DegS influences antioxidant capacity through σ^E^, we constructed a *V. cholerae rpoE* deletion mutant (*ΔrpoE*), and a double knockout strain *ΔdegSΔrseA* to revert to σ^E^ activity. qRT-PCR showed significantly reduced *rpoE* gene transcript levels in *ΔdegS* than in the WT strain, and the *ΔdegSΔrseA* strain could revert to *rpoE* levels close to those of WT (**Figure 2A**). The diameter of the inhibition zone of the *ΔrpoE* strain was significantly greater than that of the WT strain under both oxidant treatments, and the diameter of the inhibition zone of the *ΔdegSΔrseA* strain was able to revert to close to that of the WT strain (**Figure 2B**). The survival rate of the *ΔrpoE* strain in the H_2_O_2_ and CHP killing assay was significantly lower than that of the WT strain, and *ΔdegSΔrseA* strain restored close to the level of the WT strain (**Figure 2C**). These results suggest that DegS may positively regulates the antioxidant capacity of *V. cholerae* partially through σ^E^.

### Regulation of antioxidant capacity of *V. cholerae* by DegS may involve the regulation of RpoS by the cAMP-CRP complex

**Figure 3:**
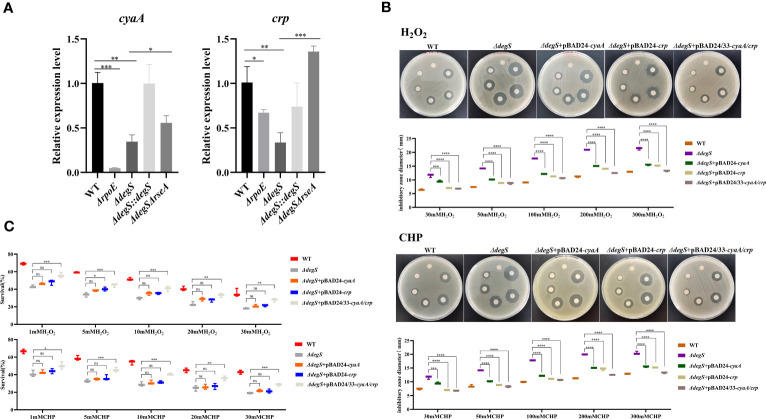
DegS regulation of antioxidant capacity in *V. cholerae* may depend on the cAMP-CRP complex. **(A)** The relative expression levels of *cyaA* and *crp* genes of different strains were compared, and 16sRNA was used for the internal reference gene (unpaired *t*-tests). **(B)** Sterile paper disk with different concentrations of H_2_O_2_ and CHP were placed on the corresponding strain plates for overnight incubation, with the oxidant concentrations ranging from small to large (0, 30, 50, 100, 200, and 300 mM) in a counterclockwise direction, and the diameter of the zone of inhibition was measured for each strain. **(C)** The killing assays were performed with 1, 5, 10, 20, and 30 mM H_2_O_2_ and CHP on the corresponding strains, and the survival rate of each strain was calculated by comparing the CFU of the treated and untreated samples. Data are means and standard deviations of three replicate experiments and were analyzed using one-way analysis of variance (ANOVA). *, *P*<0.05; **, *P*<0.01; ***, *P*<0.001; ****, *P*<0.0001, ns indicates no statistical significance.

Previous studies have shown that the cAMP-CRP-RpoS pathway plays a vital role in bacterial oxidative stress (Cheng and Sun, 2009). Gene-Act network analysis showed that the cAMP-CRP-RpoS pathway was significantly inhibited in *degS* mutants (Huang et al., 2019). CyaA encodes adenylate cyclase, which uses ATP as a substrate to synthesize cAMP, and *crp* encodes the cAMP receptor protein (CRP) (Emmer et al., 1970). qRT-PCR data showed that DegS and σ^E^ knockouts suppressed *cyaA* and *crp* expression at the transcriptional level (**Figure 3A**). To explore whether DegS regulates the antioxidant capacity of *V. cholerae* via cAMP and CRP, *ΔdegS*+pBAD24-*cyaA*, *ΔdegS*+pBAD24-*crp* and *ΔdegS*+pBAD24/33-*cyaA/crp* strains were constructed for H_2_O_2_ and CHP disk diffusion and killing assays. The results showed that *ΔdegS*+pBAD24-*cyaA* and *ΔdegS*+pBAD24-*crp* strains only partially recovered their inhibition zone diameter compared with *ΔdegS* strains, and, importantly, *ΔdegS*+pBAD24/33-*cyaA/crp* mostly recovered their inhibition zone diameter compared with *ΔdegS* strains (**Figure 3B**). In the killing assays, *ΔdegS*+pBAD24-*cyaA* and *ΔdegS*+pBAD24-*crp* were unable to revert survival compared with the *ΔdegS* strain, whereas the *ΔdegS*+pBAD24/33-*cyaA/crp* strain mostly reverted survival (**Figure 3C**). These results suggest that the regulation of antioxidant capacity by DegS in *V. cholerae* involves the synergistic action with cAMP-CRP complex.

**Figure 4:**
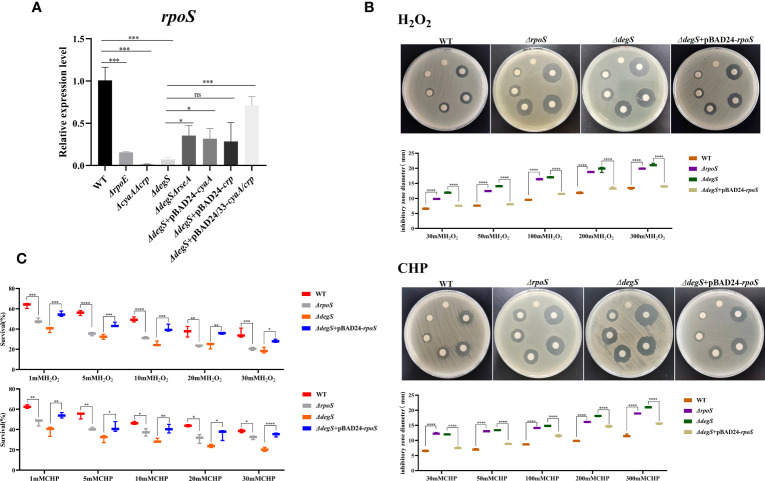
DegS regulation of antioxidant capacity in *V. cholerae* may depend on the regulation of RpoS by the cAMP-CRP complex. **(A)** The relative expression levels of *rpoS* gene of different strains were compared, and 16sRNA was used for the internal reference gene (unpaired *t*-tests). **(B)** Sterile paper disk with different concentrations of H_2_O_2_ and CHP were placed on the corresponding strain plates for overnight incubation, with the oxidant concentrations ranging from small to large (0, 30, 50, 100, 200, 300) in a counterclockwise direction, and the diameter of the zone of inhibition was measured for each strain. **(C)** The killing assay were performed with 1, 5, 10, 20, 30 mM H_2_O_2_ and CHP on the corresponding strains, and the survival rate of each strain was calculated by comparing the CFU of the treated and untreated samples. Data are means and standard deviations of three replicate experiments and were analyzed using one-way analysis of variance (ANOVA). *, *P*<0.05; **, *P*<0.01; ***, *P*<0.001; ****, *P*<0.0001, ns indicates no statistical significance.

*V. cholerae* with *rpoS* knockout is more sensitive to oxidative stress (Wurm et al., 2017). The cAMP-CRP complex is a transcriptional activator of *rpoS* (Guo et al., 2015). Our qRT-PCR experiments showed that DegS, σ^E^, cAMP and CRP affected *rpoS* transcription (**Figure 4A**). To explore whether DegS antioxidation is dependent on RpoS, we constructed a *rpoS* gene deletion strain (*ΔrpoS*) and restored *rpoS* overexpression in *ΔdegS* strain (*ΔdegS*+pBAD24-*rpoS*). Compared with the WT, *ΔrpoS* showed an enlarged inhibition zone diameter in the disk diffusion assay and a significantly reduced survival rate in the killing assay (**Figures 4B, C**). Compared with the *ΔdegS* strain, *ΔdegS*+pBAD24-*rpoS* strain showed a reduced inhibition zone diameter in the disk diffusion assay and a significantly increased survival rate in the killing assay (**Figures 4B, C**). These results suggest that DegS enhances the antioxidant capacity of *V. cholerae* through positive regulation of RpoS transcription by cAMP-CRP.

### Regulation of antioxidant capacity of *V. cholerae* by DegS involves the expression of XthA

**Figure 5:**
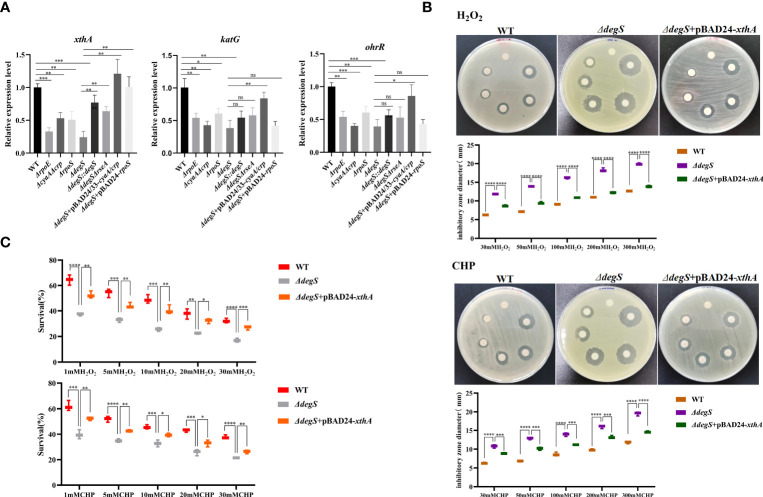
Regulation of antioxidant capacity of *V. cholerae* by DegS is related to XthA. **(A)** The relative expression levels of *xthA*, *katG*, and *ohrR* genes in different strains were compared, using 16sRNA as an internal reference gene (unpaired *t*-test). **(B)** Sterile paper disk with different concentrations of H_2_O_2_ and CHP were placed on the corresponding strain plates for overnight incubation, with the oxidant concentrations ranging from small to large (0, 30, 50, 100, 200, and 300 mM) in a counterclockwise direction, and the diameter of the zone of inhibition was measured for each strain. **(C)** The killing assays were performed with 1, 5, 10, 20, and 30 mM H_2_O_2_ and CHP on the corresponding strains, and the survival rate of each strain was calculated by comparing the CFU of the treated and untreated samples. Data are means and standard deviations of three replicate experiments and were analyzed using one-way analysis of variance (ANOVA). *, *P*<0.05; **, *P*<0.01; ***, *P*<0.001; ****, *P*<0.000; ns, no statistical significance.

We used qRT-PCR to screen genes with antioxidant capacity regulated by RpoS, including the nucleic acid exonuclease III *xthA* and catalase *katG* genes (Barth et al., 2009; Meireles et al., 2022). Moreover, the organic hydroperoxide stress resistance regulator OhrR was also examined (Barth et al., 2009; Meireles et al., 2022). These results showed that *xthA* gene transcript levels were downregulated in *ΔdegS, ΔrpoE, ΔcyaAΔcrp*, and *ΔrpoS* strains compared with WT, while *xthA* gene transcript levels were mostly reverted in *ΔdegS::degS*, *ΔdegSΔrseA*, *ΔdegS*+pBAD24/33-*cyaA/crp*, and *ΔdegS*+pBAD24-*rpoS* strains compared with WT (**Figure 5A**). The *katG* and *ohrR* gene transcript levels were downregulated in the *ΔdegS*, *ΔrpoE*, *ΔcyaAΔcrp*, and *ΔrpoS* strains compared with the WT, and partially reverted in *ΔdegS*+pBAD24/33-*cyaA/crp*, but not in the *ΔdegSΔrseA* and *ΔdegS*+pBAD24-*rpoS* strains (**Figure 5A**). Therefore, we speculated that *rpoS* exerts its antioxidant function in DegS by regulating *xthA* at the transcriptional level. Therefore, we overexpressed *xthA* in the *ΔdegS* mutant (*ΔdegS*+pBAD24-*xthA*). Compared with the *ΔdegS* strain, the inhibition zone diameter of the *ΔdegS*+pBAD24-*xthA* strain decreased (**Figure 5B**), and the killing survival rate of the *ΔdegS*+pBAD24-*xthA* strain significantly increased (**Figure 5C**). Taken together, these results suggest that regulation of the antioxidant capacity of *V. cholerae* by DegS involves XthA expression.

### DegS contributes to *V. cholerae* adaptation to starvation stress

**Figure 6:**
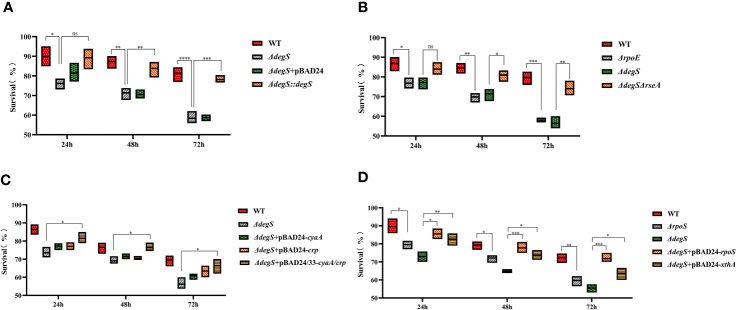
Effect of DegS on *V. cholerae* adaptation to starvation stress. **(A-D)** The bacteria were collected after anaerobic culture of the different strains and suspended in artificial seawater for 24, 48, and 72 hours by serial dilution and the number of viable cells was counted. Data are means and standard deviations of three replicate experiments and were analyzed using one-way analysis of variance (ANOVA). *, *P*<0.05; **, *P*<0.01; ***, *P*<0.001; ****, *P*<0.0001; ns, no statistical significance.

Encountering a lack of nutrients in aquatic environments is an environmental stress that *V.cholerae* regularly encounters in natural ecosystems. Therefore, it is very important for *V.cholerae* adapt to the starvation stress. To explore whether DegS helps *V.cholerae* cope with starvation stress, we tested whether DegS is important for *V.cholerae* survival in artificial seawater. To imitate the process of *V.cholerae* starvation stress, we cultured the WT strain and mutants under oxygen-limited conditions at 37°C, and then transferred the bacterial cells to artificial seawater without a carbon source at 22°C. The *ΔdegS* strain showed a significantly lower survival rate than the WT strain (**Figure 6A**). The *ΔdegS::degS* strain showed a significantly higher survival rate than that of the *ΔdegS* strain. The *ΔdegS*+pBAD24 strain exhibited phenotypic characteristics similar to those of the *ΔdegS* strain. The *ΔrpoE* strain showed a significantly lower survival rate compared to the WT, and *ΔdegSΔrseA* showed a survival rate close to that of the WT (**Figure 6B**). Compared with *ΔdegS*, *ΔdegS*+pBAD24-*cyaA* and *ΔdegS*+pBAD24-*crp* showed no significant increase in survival rate. However, the *ΔdegS*+pBAD24/33-*cyaA/crp* strain showed a significant increase in survival compared with the *ΔdegS* strain (**Figure 6C**). The survival rate of the *ΔrpoS* strain was significantly lower than that of the WT. The survival rate of *ΔdegS*+pBAD24-*rpoS* and *ΔdegS*+pBAD24-*xthA* were both significantly higher than that of *ΔdegS* (**Figure 6D**). These findings indicate that DegS is important in alleviating low levels of starvation stress in the environment, and that this role correlates with σ^E^, cAMP-CRP, and RpoS.

### DegS affects intestinal colonization ability of *V.cholerae* with or without the antioxidant

**Figure 7:**
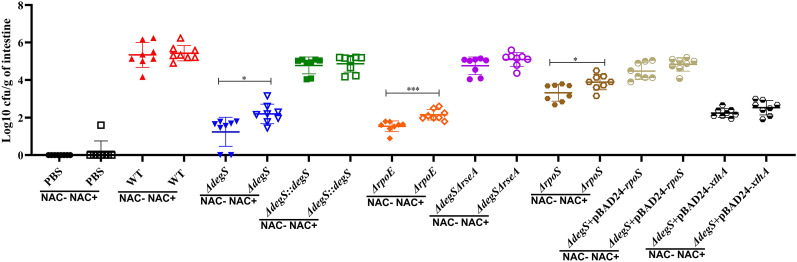
DegS affects the ability of *V. cholerae* to colonize the intestine under oxidative stress. Approximately 10^7^ cells of the different strains were inoculated by gavage in 5-6-week-old CD1 mice and incubated for 18 hours. The logarithm of colony-forming units per gram of intestine was detected by serial dilution and LB agar plates (CFU/g; mean± SD, n = 8). The data were analyzed by unpaired *t* test. *, *P*<0.05; ***, *P*<0.001.

To verify the effect of DegS on the intestinal colonization ability of *V. cholerae* under different oxidative stresses, the use of streptomycin allows for some oxidative stress in the mice intestine and promotes better colonization of the mice gut by *V. cholerae* (Spees et al., 2013; Liu et al., 2016), and employed N-acetylcysteine (NAC) as an antioxidant to reduce intestinal oxidative stress levels (Amrouche-Mekkioui and Djerdjouri, 2012). The mice were divided into two groups: a non-antioxidant group (NAC-) and an antioxidant group (NAC+). The results showed that in the NAC+ group, the *ΔdegS*, *ΔrpoE*, and *ΔrpoS* strains showed a significant decrease in colonization capacity compared with the WT strain (**Figure 7**). Interestingly, the colonization ability of *ΔdegS*, *ΔrpoE*, and *ΔrpoS* strains in NAC-treated mice was significantly higher than that of the untreated group. Compared with the *ΔdegS* strain, the colonization ability of the *ΔdegS::degS*, *ΔdegSΔrseA*, *ΔdegS*+pBAD24-*rpoS*, and *ΔdegS*+pBAD24-*xthA* strains was partially restored. In addition, we found no significant difference in the colonization ability of WT, *ΔdegS::degS*, *ΔdegSΔrseA*, *ΔdegS*+pBAD24-*rpoS*, and *ΔdegS*+pBAD24-*xthA* strains between the NAC- and NAC+ groups of mice. These results suggest that σ^E^, RpoS, and XthA are involved in DegS-mediated *V. cholerae* colonization under different oxidative stress conditions.

## Discussion

**Figure 8:**
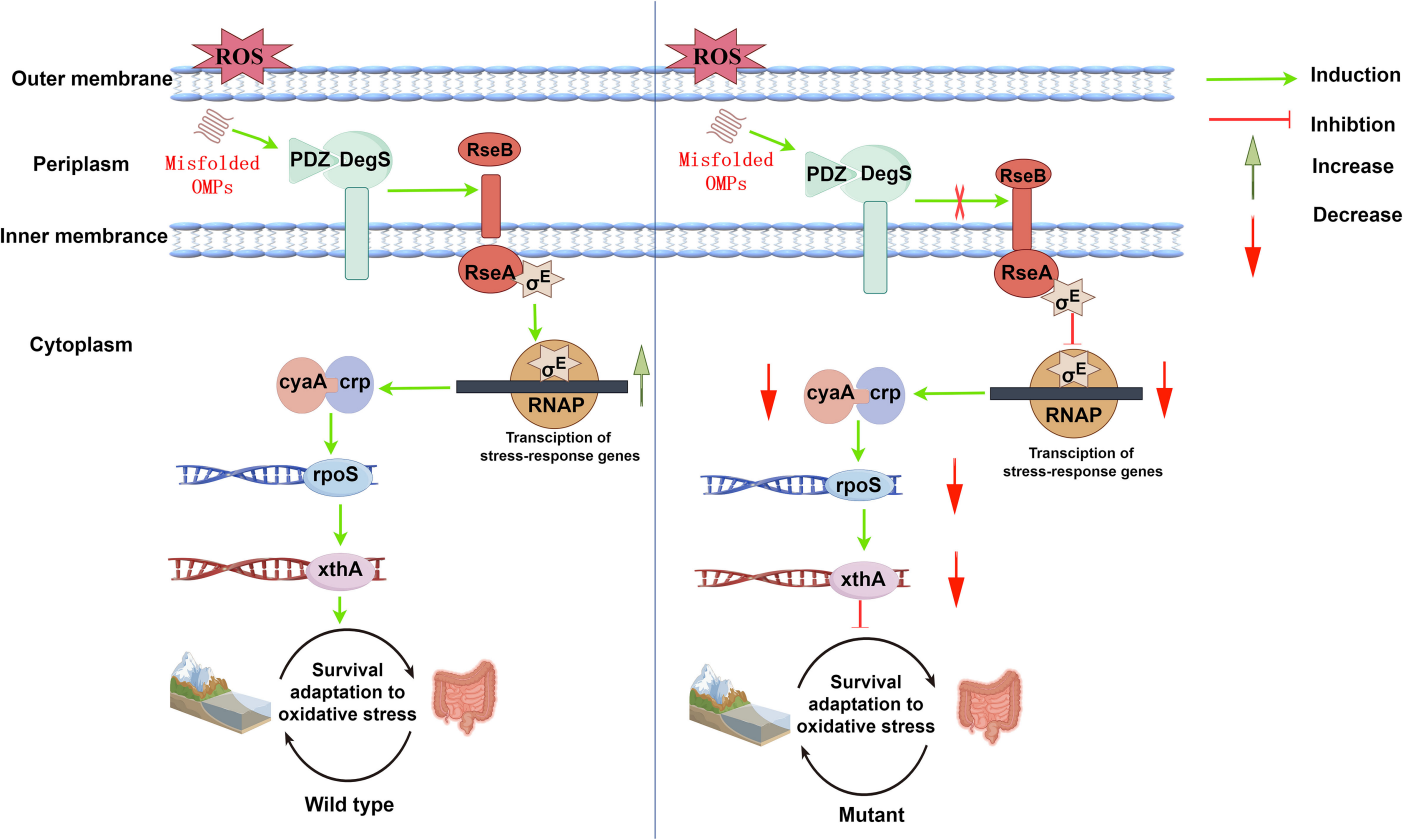
A proposed model showing the process by which DegS positively regulates *V.cholerae* antioxidant capacity and adaptation to oxidative stress environments. Under oxidative stress (wild type), the PDZ domain of DegS binds to a large number of misfolded outer membrane proteins and promotes RseA cleavage to release active σ^E^. Subsequently, *xthA* expression is induced via the cAMP-CRP-RpoS signaling pathway, initiating bacterial oxidative damage repair. Under oxidative stress (*degS* gene deletion mutation), RseA cleavage is inhibited, the number of σ^E^ and cAMP-CRP-RpoS responsive protein complexes is reduced, and *rpoS* and *xthA* transcription is down-regulated, resulting in the blockage of initiating oxidative damage repair, and a decrease in antioxidant capacity and adaptation to the oxidative stress environment. Red arrows indicate inhibition, and downward-pointing red arrows indicate reduction. Green arrows indicate induction, and upward-pointing green arrows indicate an increase.

Adapting to oxidative stress is crucial for the survival of *V. cholerae*, both in the aquatic ecosystem and within the host. When a pathogen infects a host, it can generate large amounts of ROS to protect itself from and eliminate the invading pathogen. Pathogens have developed antioxidant mechanisms to resist the deleterious effects of ROS. DegS is an influential factor in the virulence of extraintestinal infections caused by *E. coli* (Redford et al., 2003). We performed GO enrichment analysis of differential genes after *degS* knockout and showed that some of the downregulated genes were involved in the oxidative stress response. In this study, we demonstrated that DegS plays a significant role in the antioxidant capacity of *V. cholerae* and proposed a model in which DegS protease influences the expression of the nucleic acid exonuclease III *xthA gene* through the cAMP-CRP-rpoS signaling pathway, and thus regulating the antioxidant capacity and adaptation to the oxidative stress environment of *V.cholerae* (**Figure 8**).

DegS, a serine protease present in bacterial periplasm, plays an important role in the σ^E^ stress response system (Kim, 2015). Bacterial sensing of external stresses leads to the production of unfolded or misfolded proteins in the outer membrane, which bind to the PDZ structural domain of DegS protease and activate DegS protease activity, hydrolyzing the anti-σ factor RseA protein, thereby releasing σ^E^ (Hasselblatt et al., 2007). σ^E^, encoded by the *rpoE* gene, is a crucial factor for the envelope stress response in *E. coli* (Barth et al., 2009). In addition, σ^E^ also involves the regulation of bacterial antioxidant capacity. *Salmonella rpoE* mutants exhibit markedly elevated vulnerability to H_2_O_2_ (Testerman et al., 2002; Nuss et al., 2013). Our study demonstrates that the antioxidant capacity of *V. cholerae* is reduced when *degS* and *rpoE* are mutated, whereas the *ΔdegSΔrseA* strain can partially restore antioxidant activity by compensating σ^E^ activity.

To further investigate the regulatory pathways through which DegS affects the antioxidant capacity of *V. cholerae*, our RNA-seq analysis suggested that the cAMP-CRP-RpoS pathway was significantly inhibited after *degS* mutation, and related studies have shown that this pathway plays an antioxidant role in *Salmonella* (Cheng and Sun, 2009). Once CRP binds to cAMP to form a cAMP-CRP complex, the altered conformation of CRP can recognize and bind to specific sequences, thereby activating or inhibiting the expression of downstream genes (Manneh-Roussel et al., 2018). In *E. coli*, more than 7% of genes are regulated by the cAMP-CRP complex (Zheng et al., 2004). For instance, cAMP-CRP controls the supply of NADH to respiratory chain redox enzymes in central carbon catabolism (glycolysis and TCA cycles) and further controls the production of hydrogen peroxide (Kashyap et al., 2017). Our data revealed that cAMP and CRP affect the transcription of antioxidant-related genes in *V. cholerae*. Impaired antioxidant activity in *degS* mutants was partially reversed by overexpression of *cyaA* and *crp*. These results suggest that DegS affects the antioxidant capacity of *V. cholerae* possibly by regulating the cAMP-CRP complex. The cAMP-CRP complex is a transcriptional activator of *rpoS* (Guo et al., 2015). RpoS is a global transcriptional regulator involved in the regulation of oxidative stress (Chiang and Schellhorn, 2012). Our results indicate that the regulation of antioxidant capacity by DegS in *V. cholerae* may involve the cAMP-CRP complex, which regulates *rpoS*.

XthA is a nucleic acid exonuclease whose gene product enables the repair of oxidatively damaged cells (Nguyen et al., 1993). XthA transcription is dependent on *rpoS* in *E. coli* (Chiang and Schellhorn, 2012). RpoS exerts its antioxidant effects through the *xthA* gene (Charoenwong et al., 2011), which plays a dominant role in oxidative stress to hypochlorous acid (HOCl) and H_2_O_2_ stress in *rpoS* high expression strains (Barth et al., 2009). Here, qRT-PCR showed that DegS, σ^E^, cAMP-CRP, and RpoS positively regulate *xthA* gene transcription. XthA overexpression partially compensates the sensitivity of *degS* mutants to H_2_O_2_ and CHP oxidants. XthA cleaves the phosphodiester backbone of DNA upstream and removes the phosphoglycolic acid residues produced by the oxidation of the ribose portion of DNA, thus helping repair oxidized bases (Demple et al., 1986; Gupta and Imlay, 2022). Based on these findings, we propose that the effect of DegS on the antioxidant capacity of *V. cholerae* through cAMP-CRP-RpoS may be related to XthA.

In the mouse intestinal colonization experiment, we divided the mice into two groups, non-antioxidant (NAC-) and antioxidant (NAC+), and used antioxidant treatment to reduce oxidative stress in the mouse intestine. The *rpoS* mutation significantly affects the ability of *V. cholerae* to colonize the intestinal tract (Merrell et al., 2000). Decreased *V. cholerae* intestinal colonization in suckling mice is caused by mutations in the *rpoE* gene (Kovacikova and Skorupski, 2002). Our findings suggest that *V. cholerae degS*, *rpoE*, and *rpoS* deletions are associated with significantly reduced resistance to oxidative stress and the ability to colonize mice intestines. In addition, all subgroups treated with NAC showed an increase in the number of colonies in the intestine compared with the untreated group, particularly in *ΔdegS*, *ΔrpoE*, and *ΔrpoS*. The above results suggest that DegS-mediated antioxidant and mouse intestinal colonization capacity of *V. cholerae* correlates possible with σ^E^ and RpoS. Compared with the NAC- group, the ability of the *ΔdegS*, *ΔrpoE*, and *ΔrpoS* strains to colonize the mouse intestine could be partially restored after the reduction of oxidative stress in the NAC+ group, but not to a level close to that of the wild-type, suggesting that adaptation to the oxidative stress environment is only a part of the *V. cholerae*’s ability to colonize the intestine. The main reason for this is that in NAC untreated, the *ΔdegS*, *ΔrpoE*, and *ΔrpoS* mutant strains significantly reduced *V. cholerae*’s ability to colonize the mice intestine, which is in agreement with our previous findings (Zou et al., 2023). However, in the present study, we also found that with and without NAC treatment, WT, *ΔdegS::degS*, *ΔdegSΔrseA*, *ΔdegS*+ pBAD24-*rpoS*, and *ΔdegS*+ pBAD24-*xthA* strains did not differ significantly in their ability to colonize the gut. Importantly, the colonization ability of the mutant strains was partially increased by NAC treatment, suggesting that the adaptation and colonization ability of *ΔdegS*, *ΔrpoE*, and *ΔrpoS* to the intestinal environment were improved to a certain extent after the reduction of the oxidative stress in the intestinal tract of the mice by NAC treatment.

The report by Davies et al. suggests that the *xthA* mutant does not affect *V. cholerae* colonization, which is inconsistent with our study. Our study showed that replenishment of the *xthA* gene in the *ΔdegS* strain by the pBAD24-*xthA* plasmid partially restored its ability to colonize the mouse intestine in the absence of NAC treatment. Compared with the NAC untreated group, the colonization ability of the *ΔdegS* strain increased after NAC treatment, while there was no significant difference in the colonization ability of the *ΔdegS* +pBAD24-*xthA* strain, suggesting that the diminished colonization ability of the *ΔdegS* strain was only partially affected by the oxidative stress environment. XthA overexpression could eliminate the influence of oxidative stress, and could not revert to the influence of other factors, including motility, chemotaxis on intestinal colonization (Zou et al., 2023).The differences with the Davies study may be due to the different strains we used and the different mice models.

## Conclusion

In this study, we demonstrated that the deletion of *degS* may attenuate the antioxidant capacity of *V. cholerae* through *rpoE*, which in turn affects *V. cholerae* survival in aquatic environments and colonization in the mice gut. In addition, our results revealed a potential mechanism that may involve the affect of XthA expression through the cAMP-CRP-RpoS pathway. In conclusion, these findings provide novel evidence for our understanding of environmental adaptation and DegS biological function in *V. cholerae* and provide novel insights into the regulation of antioxidant activity in *V. cholerae*. However, the regulation of cAMP-CRP-RpoS and XthA by DegS-σ^E^ requires further investigation.

## Data availability statement

The datasets presented in this study can be found in online repositories. The names of the repository/repositories and accession number(s) can be found in the article/**Supplementary Material**.

## Ethics statement

The animal study was approved by Ethics Committee of the affiliated Hospital of Zunyi Medical University. The study was conducted in accordance with the local legislation and institutional requirements.

## Author contributions

KW: Writing – original draft, Writing – review & editing, Data curation, Formal Analysis, Methodology. HL: Writing – review & editing, Data curation, Formal Analysis, Methodology. MZ: Data curation, Writing – review & editing, Formal Analysis, Methodology. GW: Writing – review & editing, Methodology, Formal Analysis. JZ: Writing – review & editing, Methodology. XH: Writing – review & editing, Methodology. FR: Methodology, Writing – review & editing. HH: Writing – review & editing, Methodology. JH: Writing – original draft, Writing – review & editing, Conceptualization, Funding acquisition, Project administration, Resources, Supervision. XM: Writing – review & editing, Conceptualization, Funding acquisition, Project administration, Resources, Supervision.
