# Supplementary material for: DegS protease regulates antioxidant capacity and adaptability to oxidative stress environment in Vibrio cholerae
Source: Front Cell Infect Microbiol. 2023 Nov 20;13:1290508. doi: 10.3389/fcimb.2023.1290508 (PMC10694293; doi:10.3389/fcimb.2023.1290508)
Supplement: Supplementary file 1 [file DataSheet_1.docx]

Supplementary Material

DegS protease regulates antioxidant capacity and adaptability to oxidative stress environment in *Vibrio cholerae*

**Kaiying Wang^1,2†^, Huifang Lu^1,2†^，Mei Zou^1,2†^, Guangli Wang^1,2^, Jiajun Zhao^1,2^，Xiaoyu Huang^1,2^，Fangyu Ren^1,2^，Huaqin Hu^1,2^，Jian Huang^1,2*^ and Xun Min^1,2*^**

*** Correspondence:** Jian Huang 81537648@qq.com； Xun Min minxunzmu@163.com.

# Supplementary Tables

**Supplementary Table 1. Bacterial strains and plasmids used in this study**

| Strain or plasmid | Genotype or feature(s) | Reference or source |
| --- | --- | --- |
| Strains |  |  |
| Non-O1/non-O139 *Vibrio cholerae* Strains |  |  |
| HN375 | Wild-type non-O1/non-O139 *V. cholerae* | Lab collections |
| *ΔdegS* | in-frame deletion of *degS* | Lab collections |
| *ΔdegS::degS* | *ΔdegS* complemented with pBAD24-*degS*, Amp^r^ | Lab collections |
| *ΔdegS*+pBAD24 | *ΔdegS* complemented with pBAD24, Amp^r^ | This study |
| *ΔrpoE* | in-frame deletion of *rpoE* | This study |
| *ΔdegSΔrseA* | in-frame deletion of *degS* and *rseA* | This study |
| *ΔrpoS* | in-frame deletion of *rpoS* | This study |
| *ΔcyaAΔcrp* | in-frame deletion of *cyaA* and *crp* | This study |
| *ΔdegS*+pBAD24-*crp* | *ΔdegS* complemented with pBAD24-*crp*, Amp^r^ | This study |
| *ΔdegS*+pBAD24-*cyaA* | *ΔdegS* complemented with pBAD24-*cyaA*, Amp^r^ | This study |
| *ΔdegS*+pBAD24/33-*cyaA*/*crp* | *ΔdegS*+pBAD24-*cyaA* complemented with pBAD33-*crp*, Amp^r^and Chl^r^ | This study |
| *ΔdegS*+pBAD24-*rpoS* | *ΔdegS* complemented with pBAD24-*rpoS*, Amp^r^ | This study |
| *ΔdegS*+pBAD24-*xthA* | *ΔdegS* complemented with pBAD24-*xthA*, Amp^r^ | This study |
| Strains |  |  |
| DH5α | F−, ø80dlacZΔM15, *Δ*(lacZYA-argF) U169 deoR, recA1, endA1, hsdR17 (rk-,mk+), phoA, supE44, ʎ-, thi-1, gyrA96, relA1 | Lab collections |
| WM3064 | thrB1004 pro thi rpsL hsdS lacZ*Δ*M15 RP4-1360 *Δ*(araBAD)567 *Δ*dapA1341::[erm pir] | Lab collections |
| Plasmids |  |  |
| pWM91 | Suicide plasmid; oriR oriT lacZ tetAR sacB, Amp^r^ | Lab collections |
| pWM91-*ΔrpoE* | pWM91 carrying upstream and downstream fragments flanking *rpoE*, Amp^r^ | This study |
| pWM91-*ΔcyaA* | pWM91 carrying upstream and downstream fragments flanking *cyaA*, Amp^r^ | This study |
| pWM91-*Δcrp* | pWM91 carrying upstream and downstream fragments flanking *crp*, Amp^r^ | This study |
| pWM91-*ΔrseA* | pWM91 carrying upstream and downstream fragments flanking *rseA*, Amp^r^ | This study |
| pWM91-*ΔrpoS* | pWM91 carrying upstream and downstream fragments flanking *rpoS*, Amp^r^ | This study |
| pBAD24 | Expression vector with araBAD promoter and rrnB T1 terminator, Amp^r^ | Lab collections |
| pBAD24-*crp* | pBAD24 expressing CRP, Amp^r^ | This study |
| pBAD24-*cyaA* | pBAD24 expressing cAMP, Amp^r^ | This study |
| pBAD24-*rpoS* | pBAD24 expressing RpoS, Amp^r^ | This study |
| pBAD24-*xthA* | pBAD24 expressing XthA, Amp^r^ | This study |
| pBAD33 | Expression vector with araC promoter and rrnB T1 terminator, Chl^r^ | Lab collections |
| pBAD33-*crp* | pBAD33 expressing CRP, Chl^r^ | This study |

**Supplementary Table 2. Primers used in this study**

| Name | Primer sequence (forward/reverse, 5’ to 3’) | Use and description |
| --- | --- | --- |
| Primers for mutant construction | | |
| *rpoE* up-F | CCGCTCGAGCGGCAATAAAGCGCACGGTT | For construction of deletion mutant of *ΔrpoE* |
| *rpoE* up-R | ACCATAGTCATTACGGAATTTGCGTCGAGCGGTCACTCCTATTGTTAT |  |
| *rpoE* down-F | ATAACAATAGGAGTGACCGCTCGACGCAAATTCCGTAATGACTATGGT |  |
| *rpoE* down-R | TCCCCCGGGTACAACAACGCCTCTGCAGACTCT |  |
| *rseA* up-F | CCCTCGAGCGAATGAACGAGCAACTGACC | For construction of deletion mutant of *ΔdegSΔrseA* |
| *rseA* up-R | CTGGGTGTTGGTGAGAAGCACTATTCTGATCCTGAGCCAATCCTAAA |  |
| *rseA* down-F | TTTAGGATTGGCTCAGGATCAGAATAGTGCTTCTCACCAA CACCCAG |  |
| *rseA* down-R | CGGGATCCGGCTCAACTCCGCCACATCC |  |
| *rpoS* up-F | CGCGGATCCGGATGAGAAGATAGCGAAG | For construction of deletion mutant of *ΔrpoS* |
| *rpoS* up-R | TCATAGCCAAGAAGCCCAACAGTAAGGAGCGGTGAAAA |  |
| *rpoS* down-F | TTTTCACCGCTCCTTACTGTTGGGCTTCTTGGCTATGA |  |
| *rpoS* down-R | CCGCTCGAGACCGAACTGCCGAATGTGT |  |
| *crp* up-F | CGCGGATCCTCAATGGACGACTACCTC | For construction of deletion mutant of *Δcrp* and *ΔcyaAΔcrp* |
| *crp* up-R | TCTTGGCGAGTGATCTTGCAACTGAACCTTTTACGA |  |
| *crp* down-F | TCGTAAAAGGTTCAGTTGCAAGATCACTCGCCAAGA |  |
| *crp* down-R | CCGCTCGAGGCACAAGTTCAGCCACAA |  |
| *cyaA* up-F | CGCGGATCCAGTATCCAGAATCACATCGC | For construction of deletion mutant of  *ΔcyaA* and *ΔcyaAΔcrp* |
| *cyaA* up-R | TTGCGAAGGCTTGGAATACTATCAGCAGAAAAATAACCG |  |
| *cyaA* down-F | CGGTTATTTTTCTGCTGATAGTATTCCAAGCCTTCGCAA |  |
| *cyaA* down-R | ATAAGAATGCGGCCGCCAGCCCACAACTGACCATC |  |
| Primers for constructs for complementation | | |
| *crp* -F | CCGGAATTCATGGTTCTAGGTAAACCTCAAACC | For cloning complete length of *crp* into pBAD24 |
| *crp* -R | CCCAAGCTTTTAGCGAGTGCCGTAAACCAC |  |
| *crp* -F | GCTCTAGAATGGTTCTAGGTAAACCTCAAACC | For cloning complete length of *crp* into pBAD33 |
| *crp* -R | CCCAAGCTTTTAGCGAGTGCCGTAAACCAC |  |
| *cyaA*-F | CCGGAATTCTTGCAGGCTTATACTCAGACC | For cloning complete length of *cyaA* into pBAD24 |
| *cyaA* -R | CCCAAGCTTTTAGGCATTGACCACTTG |  |
| *rpoS*-F | CGGAATTCATGAGTGTCAGCAATACCGTAACCAAAGT | For cloning complete length of *rpoS* into pBAD24 |
| *rpoS*-R | GCGAAGCTTTTAGTTGTCGTATTCGACGTTAAACAGC |  |
| *xthA*-F | CGGAATTCATGAAAGTTATCAGCTTCAACATAAATG | For cloning complete length of *xthA* into pBAD24 |
| *xthA*-R | GCTCTAGATTACTTGAATGTTGTCCAGATAGGC |  |
| Primers for Real time PCR | | |
| q*-xthA* *-*F | TCAAAGTCCATGATGAGGCGTTTCC | For qRT-PCR to targeting *xthA* |
| q*-xthA* *-*R | CACTTCGACAGGCGTTTGTTTGC |  |
| q-*katG-*F | CGGCAAGCGGTCTAAGCATCAG | For qRT-PCR to targeting *katG* |
| q*-katG-*R | TTTGTGGAGCGAGGCGAATACG |  |
| q*-ohrR-*F | GCAATGGAGTAGTCAGCACCGAAG | For qRT-PCR to targeting *ohrR* |
| q*-ohrR-*R | AAGCAAGCAGCATAGCCGACTG |  |
| q*-rpoS-*F | CACCTGCATGCGCTTTACG | For qRT-PCR to targeting *rpoS* |
| q*-rpoS-*R | ACGCAGCATCTTAGTGACATCATCG |  |
| q-16sRNA-F | CGGTAATACGGAGGGTGCAA | For qRT-PCR to targeting 16sRNA |
| q-16sRNA-R | CACCTGCATGCGCTTTACG |  |

**Supplementary Figure 1.Relatively stable expression of 16sRNA**


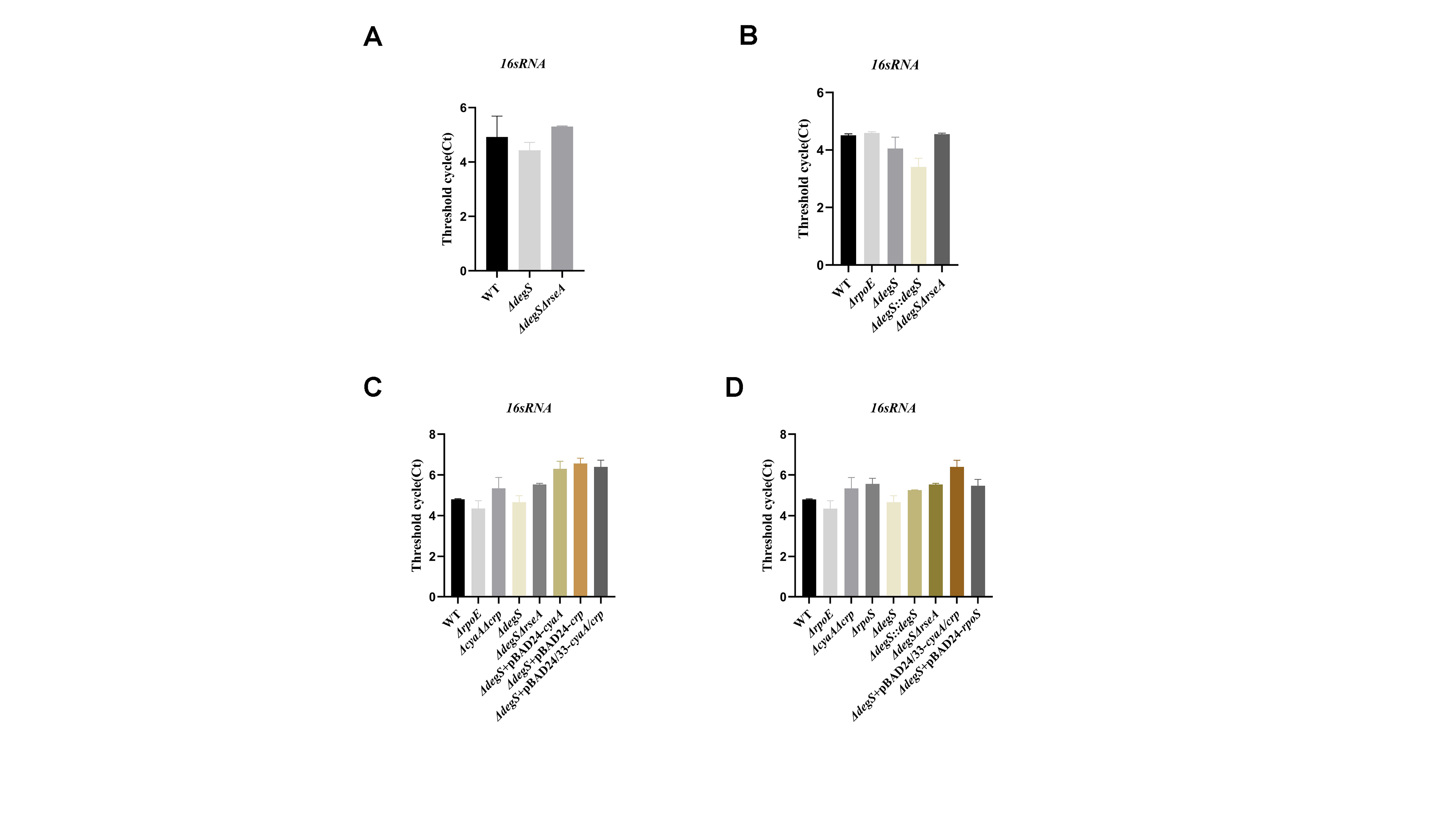


Supplementary Figure 1. (A-D) Threshold cycle (Ct) of 16sRNA expression
